# More histologic and ultrastructural degenerative signs in the subscapularis tendon and the joint capsule in male patients with shoulder impingement

**DOI:** 10.1007/s00167-017-4442-9

**Published:** 2017-03-02

**Authors:** Stefanos Farfaras, Lars Erik Ejerhed, Erling K. Hallström, Kjell Hultenby, Khaled Meknas, Tomas Movin, Nikos Papadogiannakis, Jüri-Toomas Kartus

**Affiliations:** 1 Department of Orthopedics, NU-Hospital Group Trollhättan/Uddevalla, Uddevalla Sjukhus, 451 Uddevalla, Sweden; 2 Division of Clinical Research Center, Department of Laboratory Medicine, Karolinska Institute Stockholm, Stockholm, Sweden; 3 Bone and Joint Research Group, Department of Orthopedics, Institute of Clinical Medicine, University Hospital North Norway, The Arctic University of Norway, Tromsø, Norway; 4 Department of Clinical Science, Karolinska Institute, Stockholm, Sweden; 5 Division of Pathology, Department of Laboratory Medicine, Karolinska Institute, Stockholm, Sweden; 6 Gothenburg University-Sahlgrenska Academy, Gothenburg, Sweden

**Keywords:** Subacromial impingement, Shoulder instability, Biopsy, Subscapularis tendon, Shoulder joint capsule, Degeneration

## Abstract

**Purpose:**

The purpose of the present study was to analyze biopsy samples from the subscapularis tendon and from the joint capsule from male patients with shoulder impingement syndrome (SAIS) and compare them with samples from male patients with post-traumatic recurrent shoulder instability. The hypothesis of the study was that patients with SAIS would have more histologic and ultrastructural degenerative changes in their subscapularis tendon and joint capsule than patients with post-traumatic recurrent shoulder instability.

**Methods:**

Male patients scheduled for surgery, with either subacromial decompression or Bankart reconstruction, were included. Four biopsies from each patient were obtained from the capsule and four from the subscapularis tendon during arthroscopic surgery. The histologic characteristics and the presence of glycosaminoglycans were assessed using the light microscope, and the ultrastructure was assessed using a transmission electron microscope.

**Results:**

Eight patients, median age 53 (45–74) years (*p* < 0.0001), were included in the impingement group, and 12 patients, median age 27 (22–48) years, were included in the instability group. The histologic assessment revealed significantly higher cellularity and total degeneration score in the capsule (*p* = 0.016 and *p* = 0.014 respectively) in patients with subacromial impingement compared with the instability patients. The corresponding finding was not made for the subscapularis tendon. The ultrastructural evaluation revealed that the instability patients had more fibrils with a large diameter (indicating less degeneration) in both the subscapularis tendon and the capsule compared with the impingement patients (*p* < 0.0001).

**Conclusion:**

Male patients with subacromial impingement have more histologic and ultrastructural degenerative changes in their shoulder compared with patients with post-traumatic recurrent shoulder instability.

**Clinical relevance:**

It appears that in patients with subacromial impingement, the whole shoulder joint is affected and not only the subacromial space. It is the opinion of the authors that intra-articular therapeutic injections could be tried more often in these patients.

**Level of evidence:**

III.

## Introduction

The subacromial impingement syndrome (SAIS) is the most common disease of the shoulder joint [2, 14, 34]. Individuals after the sixth decade of life are more susceptible to developing the syndrome [31]. Moreover, overhead athletes and workers, with repetitive overhead movements, develop the syndrome more frequently. Even though its prevalence is high, the etiology of this syndrome and the histologic and ultrastructural changes in the rotator cuff and the capsule are not well known. The friction and pressure in the narrow subacromial space are possibly caused by a curved or hook-shaped acromion and result in microtrauma to and sometimes inflammation in the rotator cuff, thereby provoking the pain. Subacromial decompression in patients not responding to conservative treatment has, therefore, been the treatment of choice for about three decades. The friction and pressure theory does not explain the appearance of SAIS in individuals with a normal, flat acromion configuration. Another theory, the intrinsic theory, has, therefore, been proposed. This theory is that the subacromial pain is multifactorial due, among other factors, to the chronic inflammation and degeneration of the rotator cuff and the subacromial bursa [6, 10, 29, 33]. With time, its thickening causes conflict and pain between the acromion and the rotator cuff. The pathophysiology of shoulder impingement, according to this theory, is similar to the tendinopathy in other joints of the body, such as the Achilles tendinopathy and tendinosis-like changes in the patellar tendon. Studies of torn rotator cuff tendons have revealed that degenerative changes also appear medially from the tear, indicating that the presence of degeneration before the tear occurs [3, 7, 8]. The purpose of the present study was to analyze biopsy samples from the subscapularis tendon and from the joint capsule (e.g., not directly adjacent to the subacromial space) from male patients with SAIS and compare them with samples from male patients with post-traumatic recurrent shoulder instability, to detect degenerative changes that might be present.

The hypothesis of the study was that patients with SAIS would have more histologic and ultrastructural degenerative changes in their subscapularis tendon and joint capsule than patients with post-traumatic recurrent shoulder instability. The authors hope that the study leads to a better understanding of the pathogenesis of the syndrome and helps clinicians to improve their treatment strategies in favor of the patients.

## Materials and methods

To reduce one cause of bias, only male patients referred from primary care units and scheduled for surgery, with either subacromial decompression or Bankart reconstruction, were eligible to participate in the study. The exclusion criteria were female gender, age <18 years, full-thickness supra- and/or infraspinatus tendon tears, and/or macroscopic intra-articular subscapularis tendon tears for the acromioplasty group and a glenoid fracture larger than a bony Bankart lesion for the patients planned for Bankart repair. All patients gave their written consent. Twenty patients were recruited to the study. The enrollment of the patients started in April 2012 and finished in June 2013. Group A consisted of eight consecutive patients with SAIS, who were scheduled for arthroscopic subacromial decompression, after having been treated conservatively for at least 3 months with NSAIDs, subacromial corticosteroid injections, and/or physical therapy. The diagnosis was determined with history and clinical tests with a positive painful arc test and positive impingement tests. None of the patients had a full-thickness rotator cuff tear, as determined preoperatively with MRI or ultrasound examination and confirmed macroscopically during arthroscopy. Group B, the control group, consisted of 12 consecutive patients with post-traumatic recurrent shoulder instability. These patients were the subject of surgical stabilization due to recurrent dislocations. All the subjects had dislocated their shoulder at least three times before referral to the orthopedic specialist. None of the control patients had macroscopic rotator cuff tears. In both groups, none of the patients had diabetes or rheumatoid arthritis or osteoarthritis as co-morbidities.

Before the arthroscopic intervention (subacromial decompression for Group A and Bankart reconstruction for Group B), a complete diagnostic arthroscopy was performed on each subject. After the diagnostic arthroscopy, four full-thickness biopsies were obtained from the cranial part of the mid-portion of the subscapularis tendon and four from the joint capsule just below the caudal part of the subscapularis tendon. The biopsy samples were harvested with an arthroscopic punch. Their size was approximately 1–2 × 1–2 mm.

### Ultrastructural evaluation using transmission electron microscopy

Specimens were collected and immediately fixed in 2% glutaraldehyde and 1% paraformaldehyde in 0.1 M sodium cacodylate buffer containing 0.1 M sucrose and 3 mM CaCl_2_ (pH 7.4) at room temperature for 30 min, followed by 24 h at 4 °C. They were then rinsed in 0.1 M sodium cacodylate buffer containing 3 mM CaCl_2 _ (pH 7.4) and post-fixed in 2% osmium tetroxide in 0.1 M sodium cacodylate buffer containing 1.5 mM CaCl_2_ (pH 7.4) at 4 °C for 2 h, then dehydrated in ethanol followed by acetone and embedded in LX-112 (Ladd, Burlington, Vt), for transverse sectioning. Ultra-thin sections (approximately 40–50 nm) were cut and contrasted with uranyl acetate followed by lead citrate and examined in a Tecnai 10 microscope (Fei Company, Eindhoven, the Netherlands) at 80 kV. From transverse-oriented specimens, two-to-four randomly selected areas were taken and the fibril diameter was measured, with an accuracy of 1 nm, on digital images using a semi-automatic measuring program at a magnification of ×101,000 (Soft Imaging System GmbH, Münster, Germany). The fibrils were grouped in intervals of 10 nm and presented as the relative distribution. 100 fibrils were analyzed in each specimen, and the mean value was calculated with an accuracy of the 1/10th of a nanometer. Two biopsy specimens from each patient were scanned; however, the fibril diameters were only measured in the biopsy with the best transverse orientation, and the other biopsy was left unmeasured.

### Histologic evaluation using the light microscope

The samples were fixed in 10% neutral-buffered formalin, embedded in paraffin, and sectioned at 4–5 μm, according to routine procedures. The sections were stained with hematoxylin and eosin (H&E) to evaluate fiber structure, cellularity, and vascularity and with Alcianblue (pH 2.5)-Periodic Acid-Schiff (AB/PAS) for the detection of glycosaminoglycan (GAG)-rich areas. A pathologist and an orthopedic surgeon examined the tendon specimens together using a light microscope (Leica DMRBE, Wetzlar, Germany).The examiners were blinded both in terms of the group of the patient and what location the biopsy was obtained from. This procedure and this evaluation system have been performed in multiple previous studies [1, 12, 20, 26–28]. Fiber structure, cellularity, vascularity, and level of GAGs were graded after examining the whole section. The number of cells was estimated in a high-power field (HPF) representative of the section. The results of the light-microscopic analysis were classified according to a semiquantitative, four-point scoring system (0–3), and the total degeneration score (TDS). In each patient, two biopsies from the tendon and two biopsies from the capsule were graded.

**Fig. 1:**
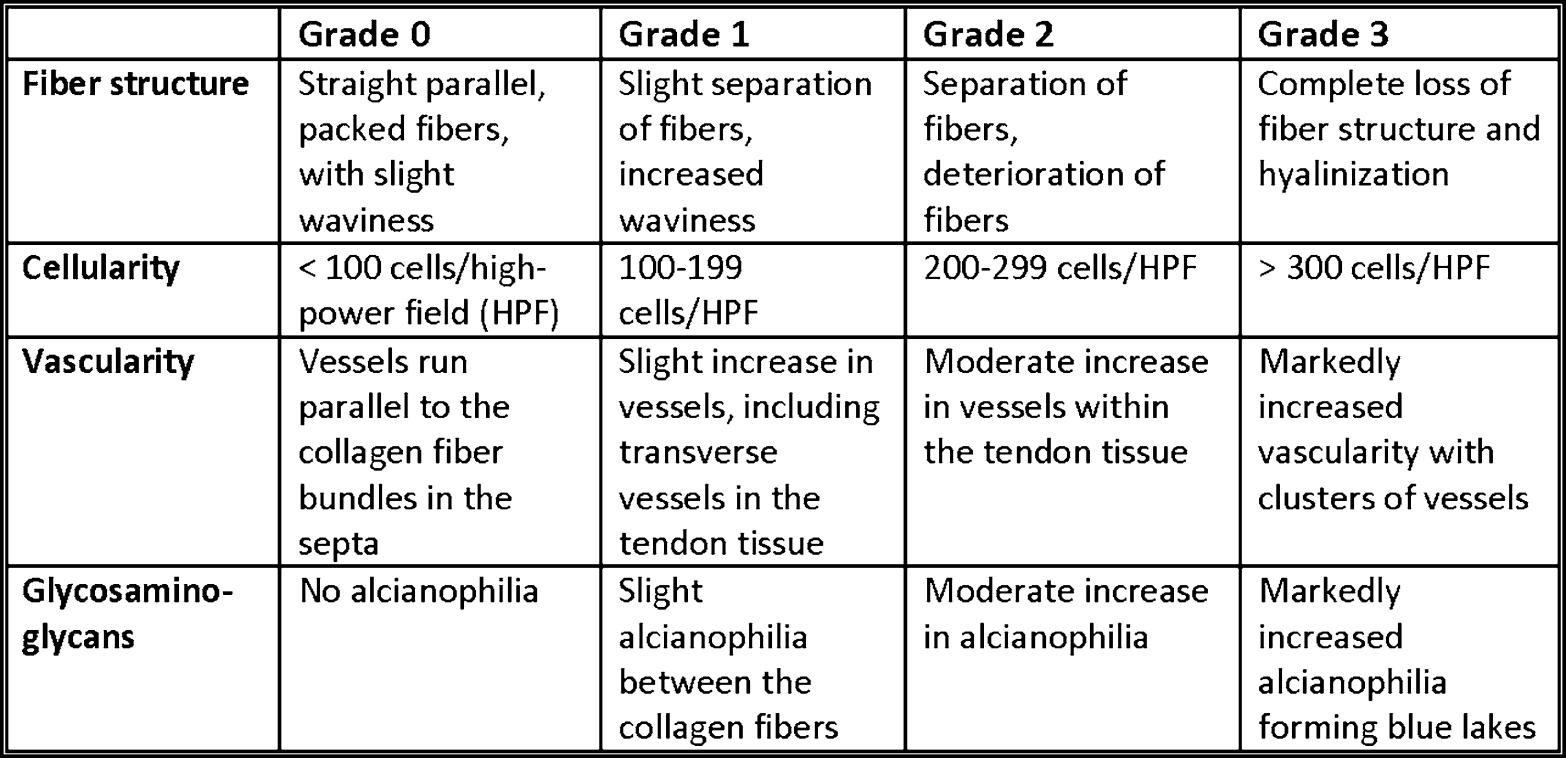
Four-point scoring system (TDS)

The TDS is similar to a scoring concept previously described by Movin et al. and used in a biopsy analysis of the Achilles tendon [22]. It consists of four different elements, such as the fiber structure, cellularity, vascularity, and GAGs. Each element can obtain between 0 and 3 points, depending on the degree of degeneration observed in the light microscope. For each sub-score, no signs of degeneration render 0 points and strong signs of degeneration 3 points. The score can result in values between 0 (no degeneration at all) and 12 points (extremely high degeneration) (Fig. 1).

The study protocol was approved by the Regional Ethical Committee, University of Gothenburg/Västra Götalandsregionen (IRB Dnr 076-12).

### Statistical analyses

Mean (SD) or median (range) values are presented when applicable. For comparisons of the fibril diameter and the TDS, the Mann–Whitney *U* was used. A *p* value of <0.05 was considered statistically significant. For the correlation analyses, the Pearson test was used. All *p* values are two-tailed.

The primary variable in the study was the fibril diameter. In the power analyses, it was estimated that a difference of 10 nm in fibril diameter between groups would be of interest to detect. To be on the safe side, it was estimated that the standard deviation would be up to four times the difference between groups. To reach a power of 80%, 252 fibril measurements from each group and from each location were required.

To increase the power of the study, 2000 (800 in the SAIS group and 1200 in the instability group) fibril diameter measurements were made from each location. This means that altogether, a total of 4000 measurements were performed.

## Results

**Fig. 2:**
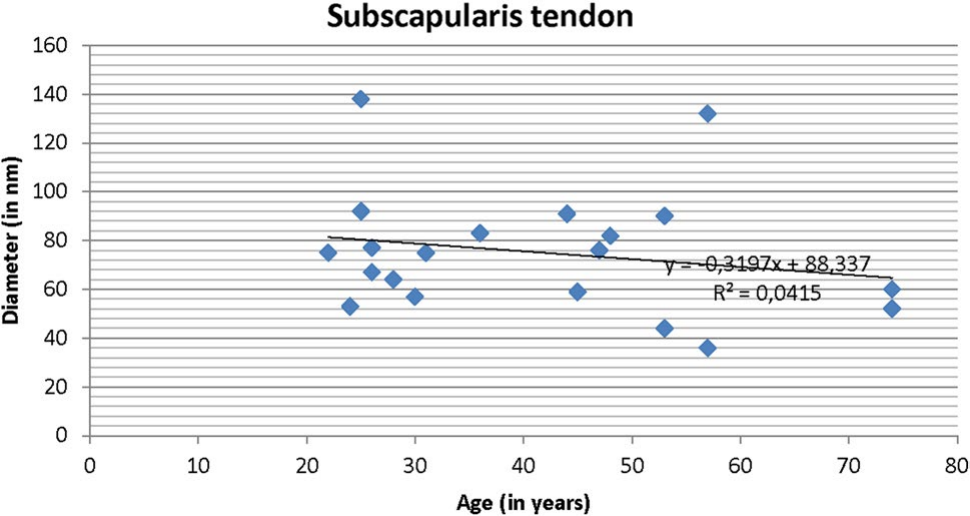
Correlation between fibril diameter and age for the subscapularis tendon

**Fig. 3:**
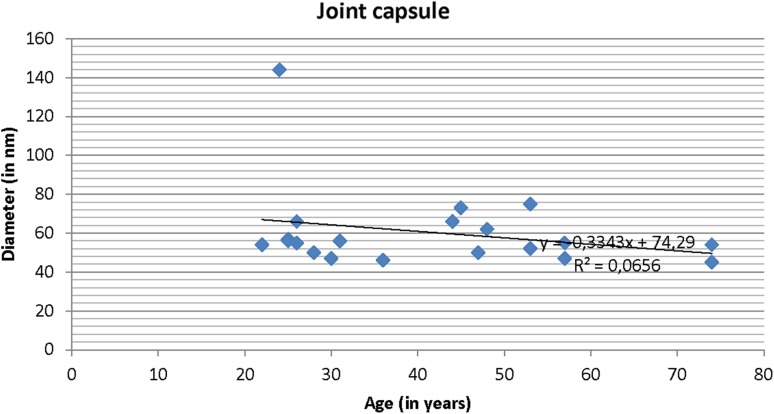
Correlation between fibril diameter and age for the joint capsule

The age distribution in the study groups is presented in Table 1. The patients in the instability group were significantly younger (*p* < 0.0001). The correlation coefficient between age and fibril diameter was *R* = −0.20 for the subscapularis tendon and *R* = −0.25 for the capsule (Figs. 2, 3).

**Table 1:** Age of the study groups

Diagnosis	Age (years)				
	Number	Mean	SD	Median	Min–max
SAIS	8	57.5	10.7	53	45–74
Shoulder instability	12	30.4	8.0	27	22–48
*p* value		<0.00001			

Patients suffering from SAIS were significantly older than patients with shoulder instability problems

*SD* standard deviation

**Fig. 4:**
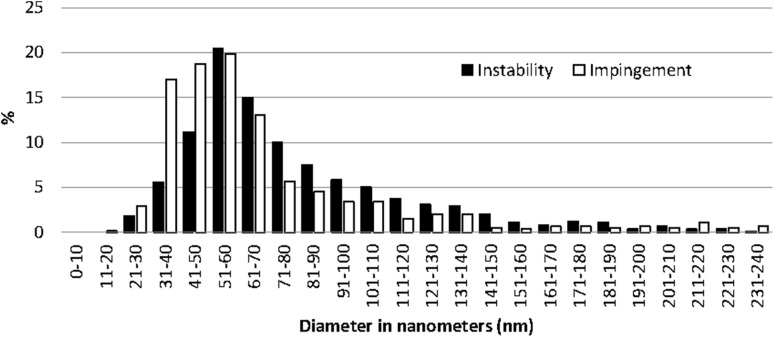
Distribution (expressed in %) of fibril diameters in the subscapularis tendon revealed that the instability patients had significantly larger fibril diameters

**Fig. 5:**
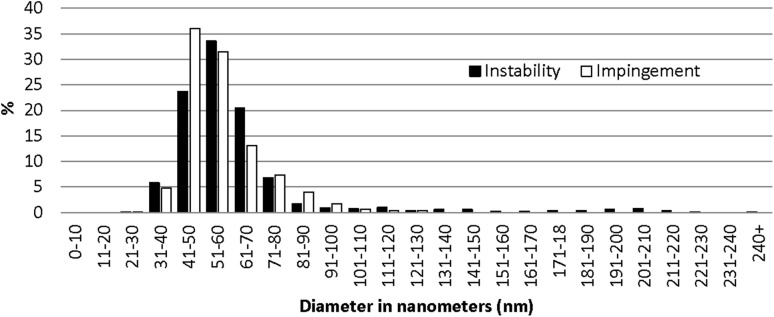
Distribution (expressed in %) of fibril diameters in the joint capsule revealed that the instability patients had significantly larger fibril diameters

The ultrastructural evaluation revealed that the instability patients had more fibrils of large diameter in both the subscapularis tendon and the capsule compared with the SAIS patients (*p* < 0.0001) (Figs. 4, 5; Table 2).

**Table 2:** Mean and median fibril diameters in the tendon and in the capsule in nm

	Tendon				Capsule			
	Mean	SD	Median	min–max	Mean	SD	Median	min–max
SAIS	68.6	40.5	57	12–240	56.3	14.2	53	30–129
Shoulder instability	79.5	38.0	67	20–233	63.2	29.6	57	30–242
*p* value	<0.0001				<0.0001			

Patients with shoulder instability had fibrils with significantly larger diameter in both the subscapularis tendon and the joint capsule compared to SAIS patients

**Fig. 6:**
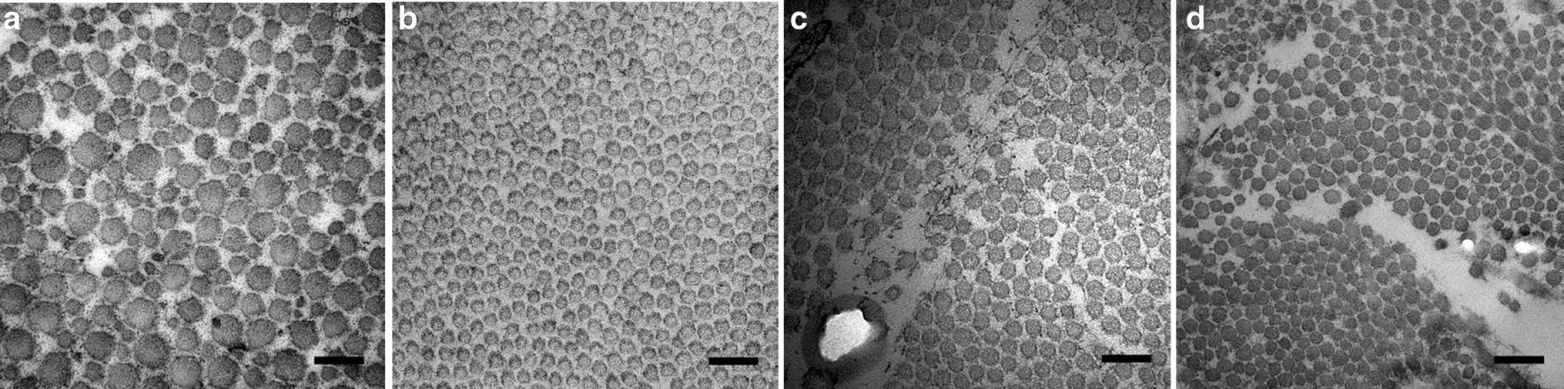
Transmission electron microscopy (TEM) images showing the fibril diameter composition in cross-sectioned subscapularis tendon (**a, b**). The fibrillar composition in the subscapularis tendon from the instability group (**a**) revealed a larger and more heterogenic fibril diameter size (mean 79.5 nm) compared with the impingement group (mean 68.6 nm). In the joint capsule, the size distribution is more homogeneous, but the diameters in the capsule of the instability group are larger (mean 63.2 nm) (**c**) compared with the impingement group (mean 56.3 nm) (**d**). *Bar* 200 nm

**Fig. 7:**
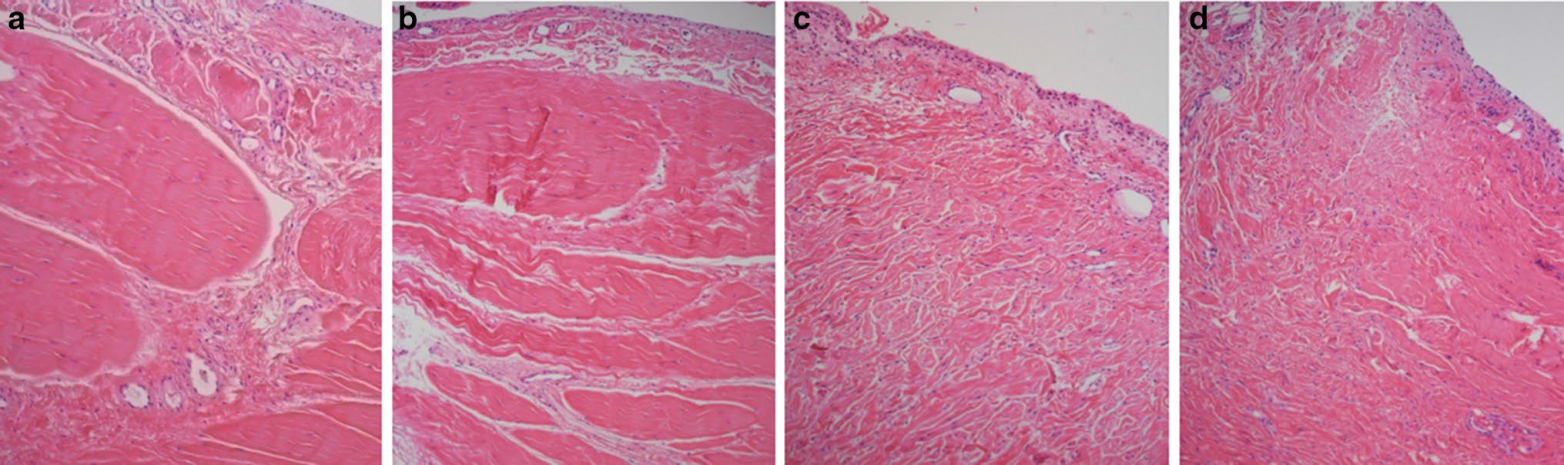
Light-microscopic views of specimens obtained from four different male patients from the subscapularis tendon (**a, b**) and the shoulder joint capsule (**c, d**). The biopsies from the instability patients are viewed in **a**, **c**, while **b** and **d** are from impingement syndrome patients. The surfaces are lined with a thin synovial layer (**a**–**d**). The subscapularis tendon (**a, b**) depicts bundles of dense connective tissue with surrounding loose connective tissue, the endotenon, containing vessels. In comparison, the joint capsule is composed of looser connective tissue with small vessels within the tissue (**c, d**).The fiber structure, cellularity, and vascularity were not significantly altered in the tendon of shoulder impingement syndrome patients, illustrated by view **b** compared with view **a**. However, signs of separation and deterioration in the fiber structure could be found in the joint capsule in both groups, while increased cellularity was more common in impingement syndrome patients, illustrated by view **d** compared with view **c**. Original magnification ×100

The cellularity was significantly higher in the capsule (*p* = 0.016), and the TDS of the capsule was significantly higher (*p* = 0.014) in patients with SAIS compared with the instability patients. The corresponding finding was not made for the subscapularis tendon (Figs. 6, 7) (Table 3).

**Table 3:** Histologic analysis according to the four-point scoring system and the total degeneration score (TDS)

Data	Capsule		Significance (*p* value)	Tendon		Significance (*p* value)
	Shoulder instability	SAIS		Shoulder instability	SAIS	
	Median (range)	Median (range)		Median (range)	Median (range)	
Fiber structure	1 (0–3)	2 (1–3)	ns (0.11)	1 (0–2)	1 (0–2)	ns (0.94)
Cellularity	1 (0–3)	1 (1–2)	0.016	0 (0–2)	1 (0–3)	ns (0.16)
Mean (SD)	0.8 (0.7)	1.3 (0.4)				
Vascularity	1.5 (1–3)	2 (1–3)	ns (0.33)	0 (0–2)	1 (0–2)	ns (0.58)
GAGs	0 (0–1)	0 (0–1)	ns (0.067)	0.5 (0–1)	1 (0–3)	ns (0.07)
TDS	4 (2–7)	5 (3–7)	0.014	2 (0–6)	3 (1–9)	ns (0.19)
Mean (SD)	4.1 (1.3)	5.1 (1.1)				

The cellularity and the TDS were significantly higher (worse) in patients with SAIS compared to patients with shoulder instability, indicating higher degeneration in the SAIS group

*ns* non-significant (*p* > 0.05), SD standard deviation, *GAGs* glycosaminoglycans, *TDS* total degeneration score

## Discussion

The most important finding of the present study was that male patients with post-traumatic recurrent shoulder instability have significantly larger fibril diameters, indicating less degeneration, in samples from both the subscapularis tendon and the joint capsule compared to male patients with SAIS. A similar pattern has been reported in tendons with tendinopathy, from other parts of the body, such as the Achilles tendon and the patellar tendon. Pingel et al. [24] showed that fibrils with a small diameter (<50 nm) are significantly increased in Achilles tendinopathy compared with healthy individuals. This finding is in line with the results of the present study, indicating that degenerative changes in the form of fibrils with smaller diameters are present in the subscapularis tendon of patients with SAIS. Likewise, Liden et al. [13] found that fibrils with sizes from 31 to 90 nm represented up to 90% of fibrils in regenerated patellar tendon, 10 years after reharvesting the patellar tendon for anterior cruciate ligament (ACL) revision reconstruction, whereas only 50% of fibrils of the same size were found in healthy control samples. Similar findings in biopsies from the patellar tendon have also been reported by Svensson et al. 6 years after primary harvest [28]. It appears that smaller fibrils are more susceptible to failure. Proctor et al. found that the repair tissue in patellar tendon in goats was composed primarily of fibrils with diameters ranging from 50 to 100 nm [25]. Furthermore, fibrils with smaller diameters (mostly between 40 and 60 nm) are found, after spontaneous rupture, in human tendons, as shown by Jozsa et al. [11]. Moreover, Magnusson et al. found significantly fewer fibrils with diameters above 60 nm in spontaneously ruptured Achilles tendons in humans [19]. These studies indicate that fibrils with smaller diameters may have poorer quality and less tolerance to load and stress. In line with this, Pingel et al. found increased numbers in cells and smaller collagen fibrils in patients with Achilles tendinopathy [24].

One possible explanation for the findings in the present study could be that patients with subacromial impingement experience a chronic process of microtrauma and regeneration, but the procedure fails to rebuild a tendon of the same quality, leading to chronic degeneration and eventually to secondary ruptures in the rotator cuff. The group with SAIS also had a significantly smaller amount of fibrils with large diameters, compared with the instability group, in their capsule samples when analyzed in the electron microscope. It is known that a traumatic rupture of the joint capsule occurs when the shoulder dislocates. Although the joint capsule in patients with recurrent shoulder dislocation is strained due to previous trauma, it appears to be in better condition compared with the capsule of patients with SAIS. The light-microscopic analysis revealed increased cellularity and increased TDS in the capsule of patients with SAIS. Findings similar to those in the present study have been reported in patients with rotator cuff tears. Longo et al. found that thinning and disorientation of the collagen fibers were more pronounced in biopsies from rotator cuff tears compared with cadaveric samples and that the pathologic score of ruptured tendons was poorer in comparison with that of the control cadaver samples [15, 16]. Pauly et al. [23] found decreased cell growth and a reduced amount of collagen type I in patients scheduled for rotator cuff repair. Both human and animal studies have demonstrated collagen cell alteration in Achilles tendinopathy. Cho et al. examined an overuse model of the Achilles tendon in rats. They found increased cellularity of fibrocytes in the right leg after 4 and 6 weeks of exercise [5]. This suggests the development of degeneration in a tendon due to overuse activity.

Furthermore, the light microscope revealed an alteration in fiber structure in the capsule in the SAIS group compared with the instability group. These patients tended to have a change in the orientation and shape of their fibers compared with patients with shoulder instability, but it was still not significant. Remarkably enough, the joint capsule appears to be in a better condition in patients with recurrent shoulder dislocation, despite the anticipated strain after shoulder dislocation. It would, therefore, have been interesting to compare the joint capsule in patients with dislocations with the capsule in healthy individuals.

As it is impossible for ethical reasons to assign healthy persons to an unnecessary operation, the use of samples from patients with recurrent shoulder instability appeared to be the most attractive way to study and compare intra-articular degenerative soft-tissue changes. An alternative control group could have been patients planned for total shoulder arthroplasty for degenerative joint disease (DJD). Such a control group would also have been “matched” in age with the SAIS group, as it is well known that DJD is common in patients above the fifth decade of life [21]. However, in such a control group, probably both the tendon and the capsule would have revealed degenerative changes in the soft tissue as well. In addition, even though the subscapularis tendon and the capsule in the control group (instability patients) can be regarded as affected samples compared with healthy tendon, significantly more degeneration was found in the SAIS group. This implies that a theoretical comparison between tendon and capsule samples from patients with SAIS and completely healthy individuals would have shown at least the same difference as the present study.

Using MRI, Gyftopoulos et al. [9] reported significant tendinosis changes in the subscapularis mid-portion and caudal portion of patients with shoulder instability compared with controls with other shoulder pathology. Moreover, the use of specimens from the subscapularis muscle as controls has previously been reported in the literature [17, 32]. Furthermore, Yuan et al. [36] have reported that there are no alterations in the subscapularis tendon, immunohistochemically, in specimens harvested from patients with recurrent shoulder dislocation.

It might be that age itself causes more degeneration. There is limited information indicating that shoulder degeneration is connected to age. Chillemi et al. found a correlation between tendon chondral metaplasia, bursal neoangiogenesis, and patient age [4]. On the other hand, signs of higher degeneration in younger patients with different tendon pathologies have been reported. Maffulli et al. [18] have examined specimens from patients with spontaneously ruptured quadriceps tendon and compared them with specimens from healthy individuals. They used a pathological score to evaluate the tendon degeneration similar to the score used in this study. Even though the patients with ruptured quadriceps tendon were significantly younger than the controls, the pathological score was significantly higher (worse) in the group of younger patients with ruptured tendons. Similar results have been reported from Tallon et al. [30] in a study examining patients undergoing surgery for Achilles tendon rupture, Achilles tendon tendinosis and individuals with no history of Achilles tendon disease. They also found fewer signs of degeneration in the control group, despite the significantly higher age. Taken into account that only a weak negative correlation between age and fibril diameter was found in the present study, it may be the case that a chronic degenerative process occurs in patients with SAIS. Further support for that the syndrome itself is more responsible than the age for the degeneration has been reported by Meknas et al. [20], who found significantly more degeneration in the obturator internus tendon in the hip in patients with osteoarthritis than in patients who had suffered a hip fracture in spite of the latter being 20 years older.

All patients in the SAIS group had subacromial corticosteroid injections administered prior to referral to surgery. This was not the case for the shoulder instability group. It appears unlikely that these injections could have affected the intra-articular space, since no patients had full thickness rotator cuff tears.

This study has several limitations. Using samples from the subscapularis tendon and capsule from patients with recurrent shoulder dislocations as controls can be questioned, since previous injury to the muscle/tendon complex and the joint capsule, due to recurrent dislocations, may exist. A further limitation is that the study was only power calculated for the difference in fibril diameter. Other limitations are that the two groups were small and had differences in terms of age and that female individuals were not examined. This was inevitable due to the instability prevalence (higher in males and younger active individuals). The main strength of the present study is that it was performed in living humans with macroscopically intact subscapularis tendons and intact capsules. Most other studies report the findings in ruptured tendons, animals or in cadaver material [3–7, 15, 17, 35].

It appears that in patients with subacromial impingement, the whole shoulder joint is affected and not only the subacromial space. It is the opinion of the authors that intra-articular therapeutic injections could be tried more often in these patients.

## Conclusion

Male patients with subacromial impingement have more histologic and ultrastructural degenerative changes in their shoulders compared with patients with post-traumatic recurrent shoulder instability.
